# Quercetin, a Natural Flavonoid Interacts with DNA, Arrests Cell Cycle and Causes Tumor Regression by Activating Mitochondrial Pathway of Apoptosis

**DOI:** 10.1038/srep24049

**Published:** 2016-04-12

**Authors:** Shikha Srivastava, Ranganatha R. Somasagara, Mahesh Hegde, Mayilaadumveettil Nishana, Satish Kumar Tadi, Mrinal Srivastava, Bibha Choudhary, Sathees C. Raghavan

**Affiliations:** 1 Department of Biochemistry, Indian Institute of Science, Bangalore-560 012, India; 2 Institute of Bioinformatics and Applied Biotechnology, Electronics City, Bangalore 560 100, India

## Abstract

Naturally occurring compounds are considered as attractive candidates for cancer treatment and prevention. Quercetin and ellagic acid are naturally occurring flavonoids abundantly seen in several fruits and vegetables. In the present study, we evaluate and compare antitumor efficacies of quercetin and ellagic acid in animal models and cancer cell lines in a comprehensive manner. We found that quercetin induced cytotoxicity in leukemic cells in a dose-dependent manner, while ellagic acid showed only limited toxicity. Besides leukemic cells, quercetin also induced cytotoxicity in breast cancer cells, however, its effect on normal cells was limited or none. Further, quercetin caused S phase arrest during cell cycle progression in tested cancer cells. Quercetin induced tumor regression in mice at a concentration 3-fold lower than ellagic acid. Importantly, administration of quercetin lead to ~5 fold increase in the life span in tumor bearing mice compared to that of untreated controls. Further, we found that quercetin interacts with DNA directly, and could be one of the mechanisms for inducing apoptosis in both, cancer cell lines and tumor tissues by activating the intrinsic pathway. Thus, our data suggests that quercetin can be further explored for its potential to be used in cancer therapeutics and combination therapy.

Among different diseases, cancer is still considered as the most detrimental for survival of humans. Chemotherapy is considered as the most promising modality for treating cancer. Although chemotherapy against cancer was introduced more than five decades ago, its application is still very limited in most of the cancers. Ideally, a chemotherapeutic drug should eradicate cancer cells by targeting a particular receptor, protein or DNA specific to the neoplastic cells and should reduce tumor burden by inducing cytotoxic and/or cytostatic effects, with least collateral damage to adjacent normal cells.

Naturally occurring compounds are considered as the most interesting agents to test for cancer prevention and therapy, due to their anticipated multimodal actions and limited toxicity. Phytochemicals may also affect the signaling pathways within the cells, including those regulating cell proliferation, activation of apoptosis etc. In addition, combined regimens of naturally occurring compounds with standard chemotherapeutic drugs are very promising in providing additive or synergistic efficacy. Among various naturally occurring compounds, polyphenols are known to be present in various edible fruits including grapes, berries, walnut, pomegranate, apples, etc. Among these, flavonoids consist of a large group of natural, small molecular weight compounds, ubiquitously present in almost all fruits and vegetables.

Polyphenols are reported to have anticarcinogenic properties against different cancers. There are several studies that address the efficiency of polyphenol containing foods in cancer prevention and therapy. However, there are only limited studies to identify individual components responsible for the anticancer properties of polyphenols. Similarly, little is known about mechanism by which such small molecule inhibitors purified from plants act on tumor cells.

Quercetin (3,3′,4′,5,7- pentahydroxy-flavone), is one of the most abundant flavonoids found in fruits and vegetables (Suppl. Fig. 1). It has been shown to exert anticancer and antiinflammatory effects^1^,^2^. Due to its antiproliferative nature and relevance in antihypertensive and neurotropic activity, it has been chemically synthesized and commercially sold^3^,^4^,^5^. There are contradicting reports on its antioxidant as well as oxidative properties^6^,^7^,^8^, which need further evaluation. The ability of quercetin to induce cell cycle arrest also warrants additional investigation as conflicting findings have been reported. For example, it appears that quercetin treatment could lead to cell cycle arrest at G0/G1 in leukemia^9^,^10^, or S phase in colorectal carcinoma^11^ or G2/M phases of the cell cycle in breast carcinoma, leukemia and oesophageal adenocarcinoma cell lines^12^,^13^,^14^. Ellagic acid is also one of the phenolic compounds abundantly present in fruits and nuts and is reported to inhibit cell mobility and cell invasion (Suppl. Fig. 1)^15^. It is known to cause cell cycle arrest at G1 phase and induce apoptosis at a concentration of 100 μM^16^.

Quercetin has been used in combination with many naturally occurring compounds like resveratrol^1^, 2-methoxyestradiol^17^, luteolin derivatives^18^, ellagic acid^2^, as well as synthetic drugs used in chemotherapy e.g., doxorubicin^19^, cisplatin^20^ etc. In several cases, combination therapy with quercetin resulted in the synergistic effects as well^1^,^17^,^21^.

In spite of various studies suggesting the cytotoxicity of quercetin and ellagic acid in various cancer cell lines, the mechanism of action of these flavonoids during tumor regression is largely unclear. Therefore, in the present study we investigated anticancer properties of quercetin in a systematic manner, using *ex vivo*, *in vitro* and *in vivo* model systems. We found that it induces several fold higher levels of cytotoxicity in cancer cells than ellagic acid. Quercetin also induced cytotoxicity in breast cancer cells apart from leukemic cells, however, its effect on normal cells was limited or none. Further, we showed that quercetin induces S phase arrest; though, we could not observe any detectable ROS production. Quercetin induced tumor regression in mice more effectively than ellagic acid and led to ~5 fold increase in survival. Quercetin induced apoptosis both in cancer cell lines and tumor tissues by activating intrinsic pathway. Thus our data indicates that quercetin could be developed further as a potential anti-cancer agent, both in conventional and combination therapy.

## Results

### Quercetin induces cytotoxicity in cancer cells

**Figure 1:**
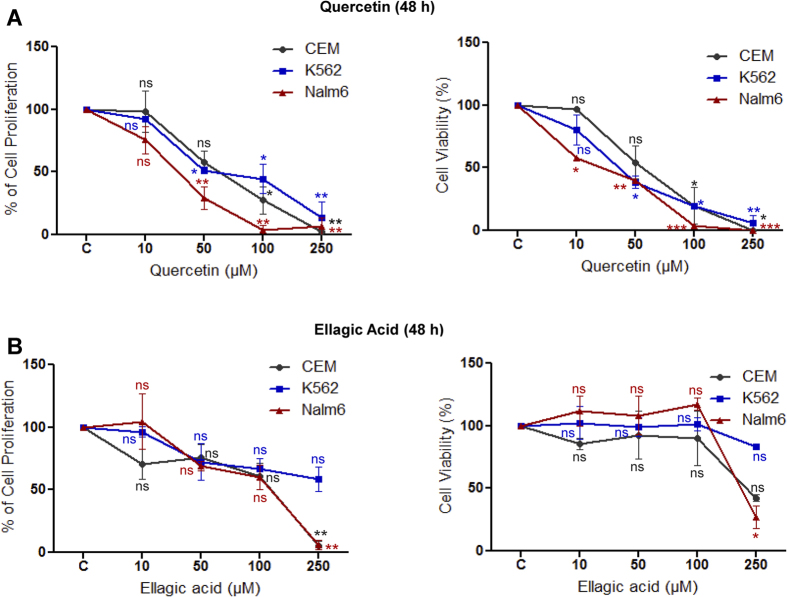
Determination of the cytotoxic effect of quercetin and ellagic acid on leukemic cell lines. Cell viability and cell proliferation was determined by MTT and trypan blue assays respectively, on CEM, K562 and Nalm6 cell lines. Cell lines were treated with quercetin (QR) (**A**) or ellagic acid (EA) (**B**) for 48 h. DMSO treated cells served as vehicle control (denoted as ‘C’) in both the cases. Concentrations of compound used were 10, 50, 100 and 250 μM. ‘ns’ is ‘not significant’ while ‘*’ represents significance (*P < 0.05, **P < 0.01, ***P < 0.001). Data represents SEM of three independent repeats.

Cytotoxic effect of quercetin and ellagic acid was examined in three leukemic cell lines (CEM, K562 and Nalm6), two breast cancer cell lines (T47D and EAC) and two normal cell lines (293T and MEF1). Cells were treated with increasing concentration of quercetin and ellagic acid (10, 50, 100 and 250 μM for 48 h) and cytotoxic effect was assessed by either MTT or trypan blue, and in some cases both. Results showed that quercetin induced cytotoxicity in all the leukemic cell lines in a dose-dependent manner while ellagic acid exhibited only limited sensitivity (Fig. 1). Among leukemic cells, Nalm6 exhibited maximum sensitivity showing reduction in cell viability even at lowest concentration of quercetin (10 μM). IC_50_ value of quercetin was estimated to be 20, 40 and 55 μM at 48 h, in Nalm6, K562 and CEM, respectively. For ellagic acid, IC_50_ was 170, >250 and 183 μM for Nalm6, K562 and CEM, respectively. Thus, our results showed that quercetin induced significantly higher toxicity in all cancer cell lines tested.

Since quercetin exhibited higher toxicity in leukemic cell lines, its effect was evaluated further in the human breast cancer cell line, T47D using MTT assay. Results showed a limited sensitivity to quercetin with an IC_50_ of 160 μM in T47D cells (Suppl. Fig. 2A). Interestingly, when quercetin was tested against mouse breast cancer cell line, EAC, it showed much higher sensitivity (IC_50_ of 50 μM), while no such effect was observed when ellagic acid was used (Suppl. Fig. 2B). However, when normal cell lines (293T and MEF-1) were analyzed using MTT assay, quercetin was found to be insensitive (Suppl. Fig. 2A). Evaluation of Nalm6 cells treated with quercetin (20 and 50 μM) by live dead cell assay following staining with Calcein-A/PI confirmed elevated cytotoxicity induced by quercetin in Nalm6 cells (Suppl. Fig. 3).

Hence, our results show that quercetin induced significant toxicity in both leukemic and breast cancer cell lines, however, its effect on normal cells was minimal.

### Quercetin induces S phase arrest during cell cycle progression followed by apoptosis

**Figure 2:**
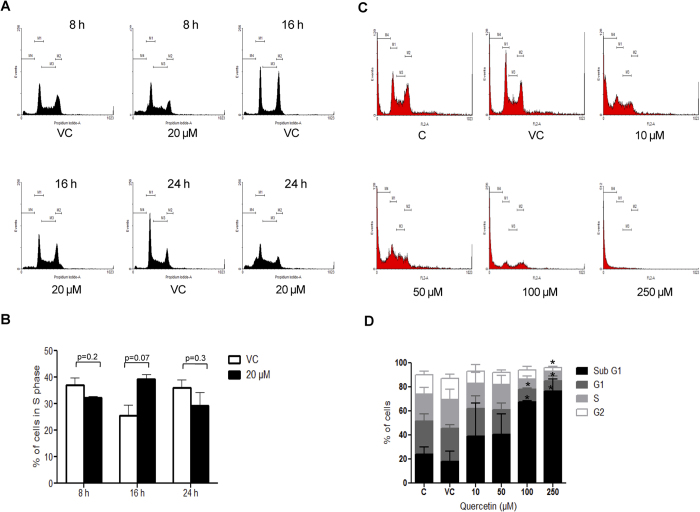
Effect of quercetin on cell cycle progression in Nalm6 cells. (**A**) Nalm6 cells (0.75 × 10^5^ cells/ml) were incubated at 37 °C with quercetin (20 μM) for 8, 16 and 24 h. Following incubation, cells were harvested, fixed and stained with propidium iodide and analysed by flow cytometry. (**B**) Bar diagram showing the percentage of cells in S phase of cell cycle. (**C**) Cell cycle distribution of Nalm6 cells after treating with increasing concentrations (10, 50, 100, 250 μM) of quercetin for 48 h. Histogram resulted after flow cytometry analysis is shown. (**D**) Bar diagrams showing percentage of cells in each phase of cell cycle. C represents cells alone, while VC stands for DMSO treated vehicle control. In each case M1 represents G1 phase, M2 represents G2 phase, M3 represents S phase and M4 represents Sub G1 phase. Error bars represents SEM of two independent repeats and ‘*’indicates the significance, *P < 0.05.

Nalm6 cells were subjected to flow cytometric analyses following treatment with quercetin (20 μM) for 8, 16 and 24 h. Results showed a distinct S phase arrest following 16 h of quercetin treatment in Nalm6 cells (Fig. 2A,B). Besides, a dose-dependent increase in apoptotic cell population was observed in Sub G1 phase when Nalm6 cells were treated with increasing concentrations of quercetin (10, 50, 100, 250 μM for 48 h) as compared to vehicle control (Fig. 2C,D). Interestingly, cell cycle arrest at S phase was observed only at lower time point (16 h) while a dose dependent increase in Sub G1 population was observed at 48 h (Fig. 2C,D). Thus, results show that quercetin induces S phase arrest followed by apoptosis in cancer cells.

### Quercetin binds to DNA by intercalation

**Figure 3:**
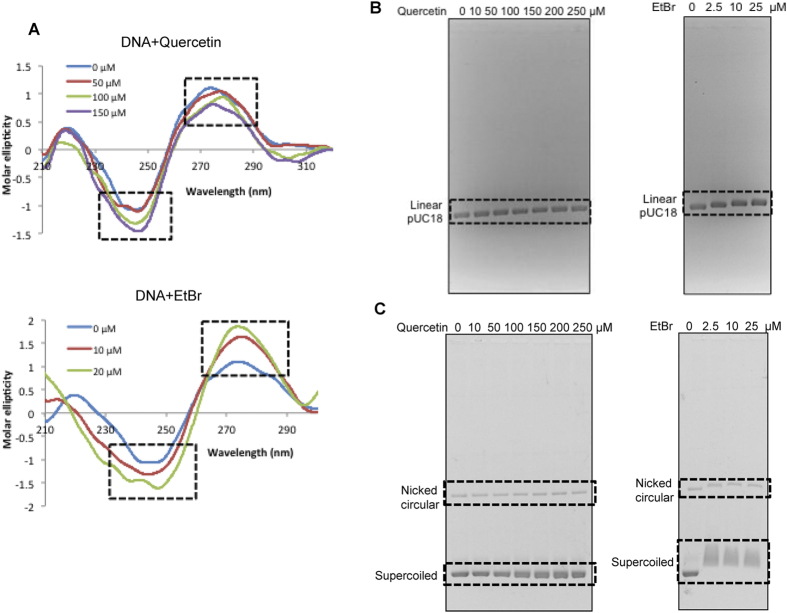
Determination of mode of action of quercetin. (**A**) Conformational changes induced by quercetin in DNA was assessed by incubating calf thymus (CT) DNA with different concentrations of quercetin (50, 100, 150 μM) for 1 h at room temperature. Following incubation, CD spectra was recorded at wavelength of 210–320 nm. Ethidium bromide (10, 20 μM) stained DNA served as a positive control. ‘0 μM’ is the untreated CT DNA. Boxed region represents the shift in DNA peak due to intercalation of the compounds. (**B**,**C**) Gel mobility shift assay was performed using 100 ng pUC18 DNA. The linearized (digested with EcoRI) (**B**) and supercoiled (**C**) pUC18 DNA were incubated at room temperature for 1 h with increasing concentrations of quercetin (10, 50, 100, 150, 200, 250 μM) and products were resolved. Ethidium bromide (2.5, 10, 25 μM) stained DNA served as positive control for the assay. Stained DNA was resolved on 1.2% agarose gel at 30 V. Boxes indicate the shift in the DNA band position due to intercalation.

DNA binding ability of quercetin was tested by incubating with calf thymus (CT) DNA, followed by CD spectroscopy. A native B-DNA conformation was observed when CT DNA was studied alone, while a negative shift in peak was observed when DNA was incubated with increasing concentrations of quercetin (50, 100, 150 μM for 1 h, RT) (Fig. 3A) suggesting an intercalation activity. Ethidium bromide stained DNA served as a positive control in which a positive shift was observed at 275 nm and a negative shift at 248 nm (Fig. 3A). Gel mobility shift assay was performed using supercoiled and linearized (digested with EcoRI) form of pUC18 DNA. A shift in the mobility of linearized pUC18 on agarose gel was observed when incubated with increasing concentrations of quercetin (10, 50, 100, 150, 200, 250 μM) (Fig. 3B). Besides, when covalently closed plasmid DNA was incubated with quercetin, a shift in mobility was observed in the case of nicked circle, while an increase in intercalation was observed for supercoiled form in a concentration (10, 50, 100, 150, 200, 250 μM) dependent manner. Ethidium bromide served as positive control for the experiment (Fig. 3C). Therefore, our data suggest that quercetin can directly interact with DNA.

### Quercetin treatment leads to significant reduction in tumor volume

**Figure 4:**
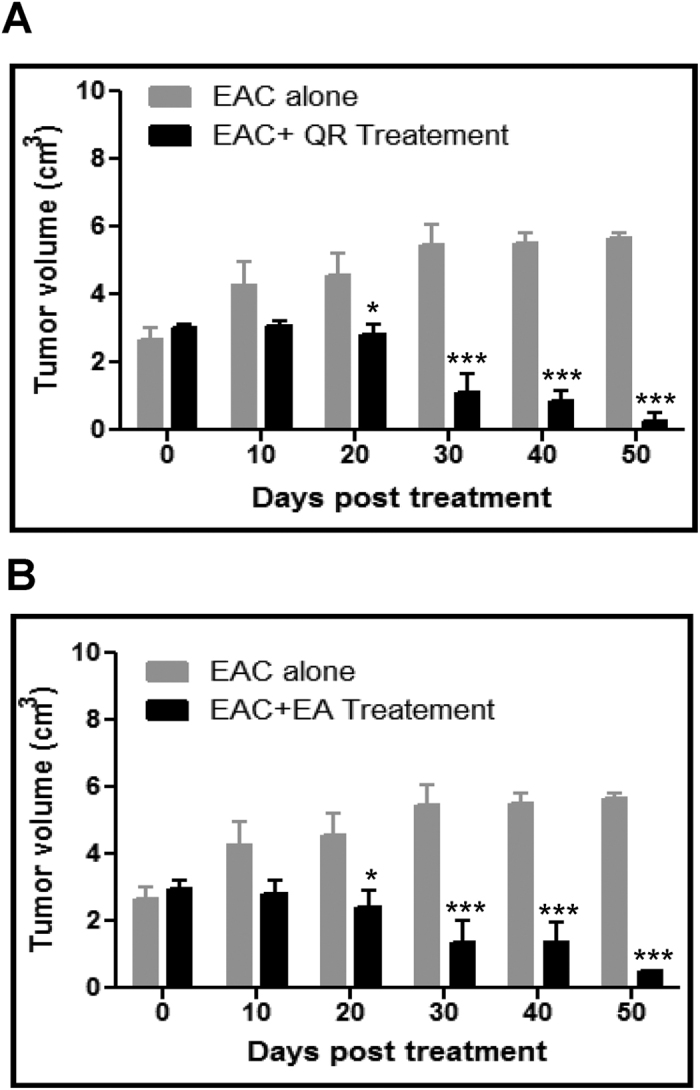
Evaluation of effect of quercetin and ellagic acid on tumor growth in mice. Solid tumor was induced in female Swiss albino mice by injecting EAC cells intramuscularly. (**A**) Six doses of quercetin (1 mg/kg body wt) and (**B**) ellagic acid (3 mg/kg body wt) were administered every third day from 12^th^ day of EAC cell injection. Data shows the tumor volume at different time intervals with and without treatment of the compound. Data was collected from three independent experiments with a set of five animals each. Error bars indicate the standard deviation (SD) of three independent experiments. P value was calculated by comparing the mean of untreated control group (EAC alone) and with mean of quercetin treated group, *P < 0.05, ***P < 0.001.

Breast adenocarcinoma developed in Swiss albino mice was used for evaluating antitumor effects of quercetin and ellagic acid. On 12^th^ day of injection of Ehrlich Ascites Carcinoma (EAC) cells, when visible tumor was detectable, mice were orally administered with 6 doses of either quercetin (1 mg/kg) or ellagic acid (3 mg/kg) on every third day. The respective doses of compounds were selected based on a pilot study (data not shown). Result showed a remarkable reduction in tumor volume when treated with each of the compounds as compared to untreated control animals bearing tumor after 10^th^ day of treatment (Fig. 4). However, the reduction in tumor size was more significant in case of quercetin treated animals, since the effect is pronounced at a dose, which is three fold lower than that of ellagic acid (Fig. 4).

### Administration of quercetin improves the lifespan of tumor bearing mice, with no side effects

**Figure 5:**
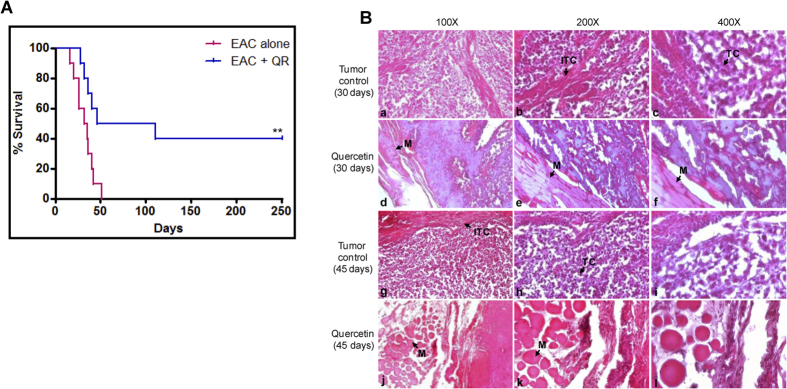
Evaluation of effect of quercetin on survival of tumor bearing mice and its side effects. (**A**) Kaplan-Meier survival curve of female Swiss albino mice treated with quercetin (1 mg/kg, six doses). The experiment was performed in two independent batches with a set of five animals each. ‘**’ represents significance, **P < 0.01. (**B**) Histopathologic analysis of tumor following quercetin treatment. Histological sections of thigh of tumor bearing mouse with (treated tumor) and without treatment (control tumor) of quercetin after 30^th^ day (d–f) and (a–c) and 45^th^ day (j–l) and (g–i) of tumor development. Final magnifications shown are 100× (a, d, g and j panel), 200× (b, e, h and k panel), and 400× (c, f, i and l panel). ‘M’ is muscle, ‘TC’ is tumor cells and ‘ITC’ stands for infiltering tumor cells.

Increase in life span was evaluated in case of quercetin treated (1 mg/kg) tumor bearing mice as described in Materials and Methods. While untreated control mice survived for a maximum of only ~50 days, treatment with quercetin led to a ~5 fold increase in the life span for atleast 40% of mice (Fig. 5A). This confirms that administration of quercetin did not result in any side effects. Thus, our data shows that quercetin treatment in mice leads to lower tumor load, increased survival and minimal side effects.

Histopathological studies were done on tumor section of control and quercetin treated mice at 30^th^ and 45^th^ days of treatment, by hematoxylin and eosin staining (Fig. 5B). High nuclear staining by hematoxylin was observed in control tumor animals (Fig. 5B; a–c) thus showing the presence of large number of proliferating cells as compared to treated tumor (Fig. 5B; d–f). While control tumor muscle at 30^th^ day of tumor development showed distorted histology and infiltration of tumor cells (Fig. 5B; a–c), following quercetin treatment, cell proliferation and damage in muscle architecture was limited (Fig. 5B; d–f). This was further reduced on 45^th^ day of quercetin treatment (Fig. 5B; j–l) in comparison to control (Fig. 5B; g–i).

**Figure 6:**
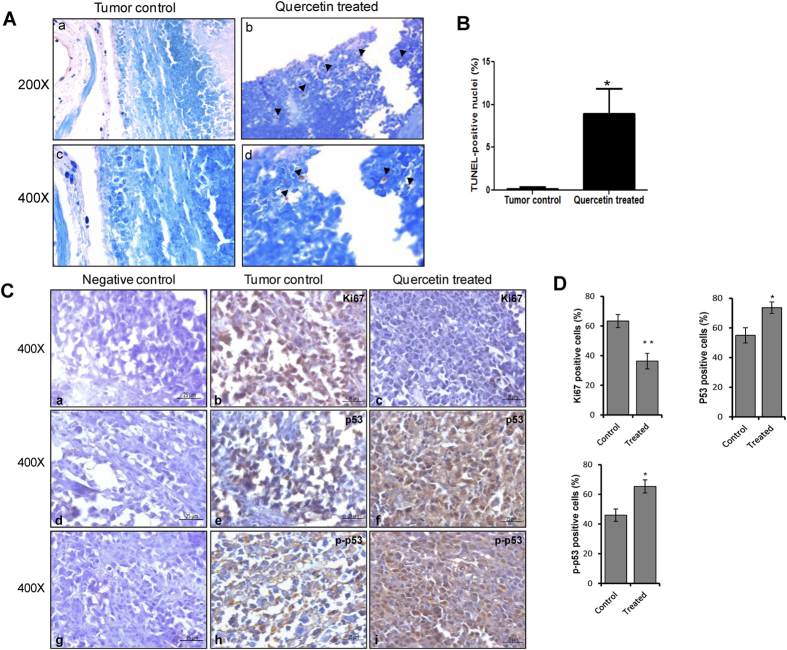
Evaluation of quercetin mediated cytotoxicity and its mechanism in tumor tissues. (**A**) TUNEL assay showing DNA fragmentation on 30^th^ day of quercetin treated tumor tissues in comparison to untreated control tumor. Brown color nuclei indicate DAB staining showing DNA breaks, while nuclei with intact DNA were stained with methyl green. Final magnifications shown are 200× and 400×. (**B**) Bar diagram with SEM (n = 5) represents % TUNEL positive nuclei in tumor control and quercetin treated mice tissues. ‘*’ represents P < 0.05. (**C**) Immuno staining was performed on paraffin embedded tissue sections for Ki-67, p-p53 and p53 using respective antibodies in control tumor tissues and quercetin treated tumor tissues after 30^th^ day of treatment. Final magnification shown is 400×. (**D**) The images of IHC were quantified and plotted as SEM (n = 5). ‘*’represents significance (*P < 0.05, **P < 0.01).

TUNEL staining was observed in quercetin treated tumor tissue section (30^th^ day) in comparison to untreated control tumor tissues (Fig. 6A,B) suggesting the presence of DNA fragmentation, which is a characteristic feature of apoptosis. Thus, our data suggest that quercetin treatment might activate apoptosis in tumor tissues.

Immunohistochemical analysis showed strong nuclear staining of Ki-67 (Fig. 6C; b), a cellular proliferation marker, in untreated tumor tissue sections (30^th^ day). In contrast, substantially less number of Ki67 positive cells were observed in quercetin treated tissue sections (Fig. 6Cc,D). Further, we observed significant increase in p53 and p-p53 (phospho-p53) positive cells in quercetin treated tumor sections (Fig. 6Cf,i,D), as compared to untreated controls (30^th^ day) (Fig. 6Ce,h) suggesting activation of apoptotic pathway. Tissue sections with no primary antibody served as negative control in each case (Fig. 6Ca,d,g).

### Quercetin treatment results in depolarization of mitochondrial membrane potential

**Figure 7:**
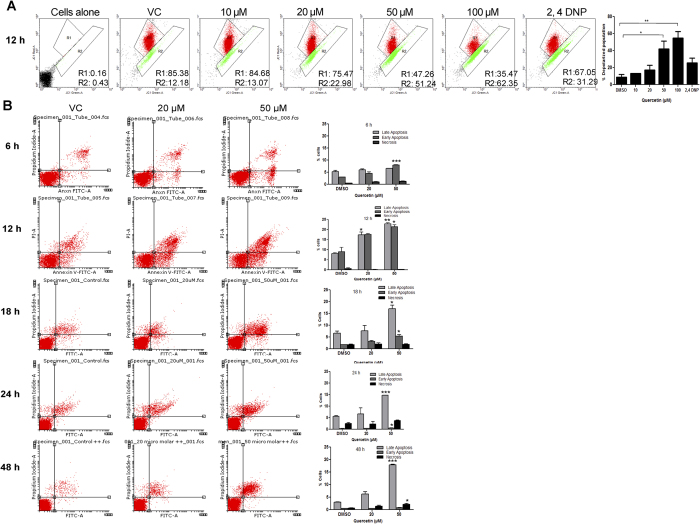
Assessment of effect of quercetin on mitochondrial transmembrane potential (ΔψM) and apoptosis. (**A**) Nalm6 cells were treated with quercetin (10, 20, 50 and 100 μM) for 12 h. Cells were harvested, stained with JC1 dye for 20 min at 37^o^ C and analyzed using flow cytometer. Depolarization in mitochondrial membrane potential was assessed by a shift from red to green fluorescence, where red indicates JC1 aggregates in intact mitochondria and green represents green fluorescing monomers in cytosol depicting loss in mitochondrial potential. VC represents DMSO treated vehicle control. Bar diagram shows percentage of depolarized population after quercetin treatment. ‘*’represents significance where *P < 0.05, **P < 0.01. 2,4 DNP treated cells served as positive control. (**B**) Annexin V/PI dual staining of Nalm6 cells for the detection of apoptotic stages following treatment with quercetin. Nalm6 cells were stained with annexin V-FITC/PI after treatment with quercetin (0, 20 and 50 μM) for 6, 12, 18, 24 and 48 h. Cells were analysed quantitatively as well as qualitatively. Histogram showing distribution of annexin V-FITC/PI stained cells. In each panel lower left quadrant shows the cells negative to both annexin V-FITC and PI staining, lower right panel shows the cells stained with annexin V (early apoptotic cells), upper left quadrant shows cells stained with PI alone (necrotic cells) and upper right shows the cells positive for both annexin V and PI (late apoptotic cells). Histogram showing distribution of early and late apoptotic cells as well as necrotic cells following treatment with quercetin for 6, 12, 18, 24 and 48 h, respectively. Error bar represents standard error mean (SEM) of atleast two independent repeats. P value has been calculated as compared to DMSO control in each case where *P < 0.05, **P < 0.01, ***P < 0.001.

Depolarization in mitochondrial membrane potential was studied by staining treated and untreated cells with JC1 dye. The depolarization leads to a shift from red to green fluorescence leading to the release of CYTOCHROME C. Interestingly, a significant shift from red to green fluorescence was observed when Nalm6 cells were treated with increasing concentrations of quercetin (10, 20, 50, 100 μM) for 12 h as compared to vehicle control. 2,4 dinitro phenol (2,4-DNP) was used as a positive control (Fig. 7A). Hence our data suggest the involvement of mitochondrial pathway of apoptosis during quercetin mediated cytotoxicity.

Accumulation of ROS after treatment with antitumor compounds is one of the factors responsible for cells undergoing DNA damage and apoptosis. We were interested in checking whether Nalm6 cells were undergoing cell death due to ROS production. In order to test this, the cells were treated with quercetin (25 μM) for different time points and subjected to flow cytometry. H_2_O_2_ induced ROS production served as a positive control. Results showed that quercetin did not induce any significant change in the levels of ROS (Suppl. Fig. 4A,B) suggesting that cell death induced by quercetin was not due to ROS production.

### Quercetin treatment leads to apoptosis

In order to confirm whether the cytotoxicity induced by quercetin is indeed due to apoptosis, rather than necrosis, annexin V-FITC/PI staining was performed following quercetin treatment. Nalm6 cells treated with quercetin (0, 20, 50 μM for 6, 12, 18, 24 and 48 h) were subjected to annexin V-FITC/PI double-staining followed by flow cytometry (Fig. 7B). Apoptotic cells can be detected by virtue of binding of annexin V-FITC to phosphotidyl serine (PS) present on the outer side of the cell membrane. In normal cells PS is predominantly present on cytosolic side of the membrane, while it gets translocated towards outer side of the membrane when cells undergo apoptosis. Results showed a concentration dependent increase in early apoptotic cells upon treatment with quercetin for 6 and 12 h, while an increase in late apoptotic cells were observed at 18, 24 and 48 h of treatment suggesting activation of apoptotic pathways (Fig. 7B). Further, confocal microscopic analysis showed cells stained with annexin V-FITC (green fluorescence), following treatment with quercetin (20 μM) suggesting cells in early apoptotic phase (Suppl. Fig. 5). In contrast, most of the cells were stained with annexin V-FITC/PI (green and red) at higher concentrations of quercetin (50 μM) indicating that these cells were undergoing late apoptosis (Suppl. Fig. 5).

### Quercetin activates intrinsic pathway of apoptosis

**Figure 8:**
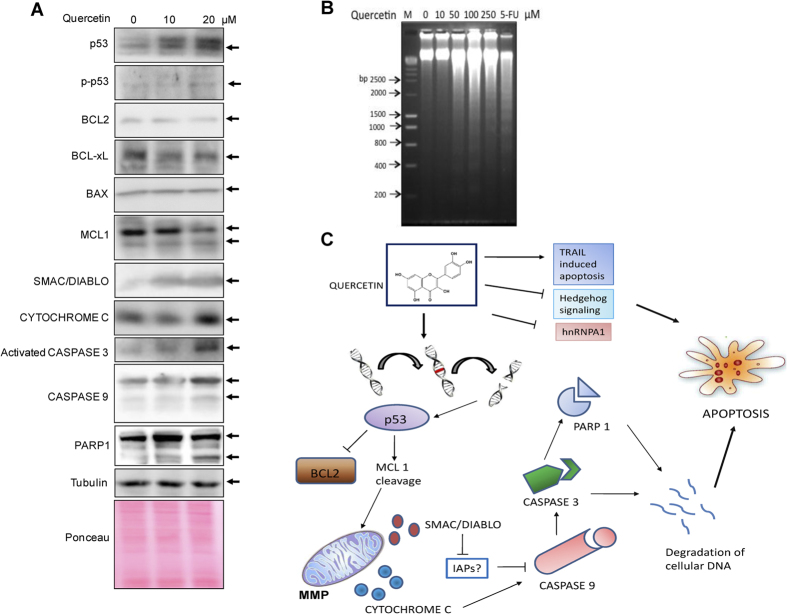
Effect of quercetin on expression of apoptotic proteins and mechanism of action. (**A**) Expression level of apoptotic proteins following treatment with quercetin. Cell lysate was prepared after treating Nalm6 cells with quercetin (0, 10, 20 μM, for 24 h). Protein lysate was resolved on SDS-PAGE and immunoblotting was performed using appropriate primary and secondary antibodies. Expression pattern was studied for p53, p-p53, BCL2, BCL-xL, BAX, MCL 1, CYTOCHROME C, SMAC/DIABLO, activated CASPASE 3, CASPASE 9, PARP1 and Tubulin. Ponceau stained PVDF membrane after protein transfer acted as a control for equal protein loading. (**B**) Detection of DNA fragmentation in Nalm6 cells following treatment with quercetin. Chromosomal DNA was extracted after treatment with quercetin (10, 50, 100, 250 μM). The purified DNA was resolved on 2% agarose gel at 50 V for 5 h. M is hyper ladder I (Bioline). 5-fluorouracil (5-FU; 100 μM) was used as positive control. (**C**) Proposed model for quercetin induced apoptosis leading to cytotoxicity. Treatment of cancer cells with quercetin leads to apoptosis through activation of mitochondrial intrinsic pathway. Since quercetin is a DNA intercalator, it might induce DNA damage, which leads to the upregulation of p53. This results in the down regulation of antiapoptotic protein, BCL2 and cleavage of MCL1. CYTOCHROME C and SMAC/DIABLO are released due to loss in mitochondrial membrane potential. SMAC/DIABLO inhibits IAPs (inhibitors of apoptosis proteins), while CYTOCHROME C release leads to cleavage of CASPASE 9, which in turn cleaves CASPASE 3. CASPASE 3 leads to fragmentation and degradation of cellular DNA and activation of PARP1 resulting in apoptosis.

In order to find out the mechanism by which quercetin induces apoptosis, western blotting studies were performed to evaluate the alterations in the protein expression. Nalm6 cells were treated with increasing concentrations of quercetin (0, 10, 20 μM for 24 h), lysate was prepared and analyzed for various apoptotic pathway proteins. Results showed an increase in level of p53 and p-p53 along with cleavage of MCL1, an apoptotic marker (Fig. 8A; Suppl. Fig. 6). A reduction in level of anti-apoptotic proteins BCL2 and BCL-xL was observed while a concomitant increase in level of BAX, a proapoptotic protein was also noted (Fig. 8A). Further, the release of CYTOCHROME C in conjunction with SMAC/DIABLO indicated the activation of mitochondrial intrinsic pathway resulting in apoptosis (Fig. 8A). The observed increase in the level of activated CASPASE 3, cleaved CASPASE 9 and PARP1 further confirmed such a conclusion (Fig. 8A). The data was quantitated, normalized with respect to tubulin and represented as bar diagram with standard error bar (Suppl. Fig. 6).

DNA fragmentation is one of the hallmarks of apoptosis differentiating between the necrotic and apoptotic modes of cell death. To test for the presence of DNA fragmentation, Nalm6 cells were treated with different concentrations of quercetin (0, 10, 50, 100, 250 μM) for 48 h. An increase in endonuclease mediated cleaved DNA ladder intensity was observed with increasing concentration of quercetin after 48 h further confirming the apoptosis (Fig. 8B). 5-fluorouracil (5-FU), a compound known for inducing DNA fragmentation was used as the positive control for the assay.

## Discussion

Development of safe, novel and effective drugs with less toxicity and lesser side effects is needed for cancer therapeutics. DNA double strand breaks, if not repaired can also lead to genomic instability and cancer. Hence targeting DSB repair genes is one of the methods for cancer therapeutics^22^. Previous studies from our lab have shown cytotoxic properties of extracts prepared from fruits such as strawberry^23^ and sapota^24^, Methyl Angolensate purified from callus of Red wood tree^25^,^26^,^27^ and many synthetic compounds like MPTQ^28^,^29^, SCR7^22^,^30^,^31^, Levamisole derivative (4a)^32^, ASHD^33^, hydantoin derivatives DFH and DCH^34^ etc. on various cancer cells. These compounds have been shown to be antiproliferative and interfered with different cellular signaling pathways to induce apoptosis.

Phytochemicals are extensively used for evaluation of their cancer therapeutic properties^35^. Quercetin and ellagic acid are two such compounds studied for their anticancerous and antiproliferative properties as single molecule or in combination^1^,^2^,^21^,^36^. These natural flavonoids are thought to act as antioxidants and provide defense against several pathologies.

Previous reports showed that both quercetin and ellagic acid induced cytotoxicity in various human cancer cell lines, with varying sensitivity^1^,^12^,^16^,^37^. In our *ex vivo* assays, we find that quercetin induces cytotoxicity in leukemic cells effectively; pre-B cell line, Nalm6 being most sensitive with an IC_50_ value of 20 μM (Fig. 1). A varying range of IC_50_ for quercetin was reported earlier^38^,^39^,^40^,^41^,^42^,^43^, which ranges from 10 μM to >100 μM. In our studies, quercetin was more cytotoxic compared to ellagic acid. Between the breast cancer cell lines tested, the one derived from mouse showed more sensitivity. However, normal cells derived from human embryonic kidney as well as mouse embryonic fibroblasts were insensitive to quercetin. The ability of quercetin to spare normal cells has also been reported previously^44^. Chronic myelogenous leukemia cell line, K562 was reported to display resistance to most of the anticancerous drugs. However, we observe that it was sensitive towards quercetin, which was consistent with a previous study^8^.

A prominent cell cycle arrest at S phase was noted upon treatment with quercetin in Nalm6 cells at early time points (16 h). Besides, an increase in cells in Sub G1 phase was observed in a dose dependent manner, when Nalm6 cells were treated for 48 h. This suggests that quercetin might induce DNA damage, which need to be corrected before cell division takes place. Considering the ability of quercetin to interact with DNA, the observation can be easily explained (see below). A similar S phase arrest has been observed previously as well^5^,^11^. However, as reported by others, we did not observe either G0/G1 or G2/M arrest^9^,^10^,^12^,^13^,^14^. Difference in the cancer type used may account for such a disparity in the observation.

Consistent with the cytotoxic effect induced by quercetin, we find that even in the tumor tissues, it induced 3-fold more cytotoxicity than ellagic acid. While an impressive, 1 mg/kg quercetin was sufficient for significant inhibition of tumor growth in breast adenocarcinoma bearing mice, for an equivalent effect we had to administer 3 mg/kg of ellagic acid. The dose at which tumor regression was observed in the current work was 10 times lower than that used in past studies^19^,^45^. This discrepancy could be due to differences in the cancer cell types or mouse strains used for developing tumor. EAC cells used in the present study are undifferentiated malignant cell lines derived from mice breast adenocarcinoma, commonly used for inducing tumors in mice and used to assess the anticancer effect of various compounds^24^,^28^,^31^,^46^,^47^,^48^. Thus we conclude that quercetin is more effective than ellagic acid in inducing cytotoxicity in different cancer cells.

A 5-fold increase in life span was observed in tumor bearing animals following treatment with quercetin compared to that of untreated tumor bearing mice. This indicates that quercetin induced cytotoxicity in tumor cells without significantly affecting the normal cells. Remarkably, at least 40% of the tumor bearing animals survived upto 250 days. Histological studies and Ki-67 staining confirmed a reduction in proliferative cells and a low damage in muscle architecture in quercetin treated tumor animals in comparison to the untreated control.

Intercalation of small molecules causes conformational changes in DNA, which can affect physiological processes such as replication, transcription, translation and repair. CD spectroscopic and gel shift studies showed that quercetin could intercalate to DNA (Fig. 3), which was also consistent with a recent report^38^. DNA intercalation is one of the mechanisms by which anticancerous drugs cause DNA damage, accumulation of which could culminate in apoptosis. The observed cell cycle arrest at S phase was also consistent with the plausible induction of DNA damage following DNA intercalation within the cells. However, we could not observe any ROS generation suggesting that it might act as an antioxidant, which was also consistent with another study^49^.

Depolarization in mitochondrial membrane potential in Nalm6 cells, TUNEL staining positive cells in tumor tissues, Annexin V/PI staining showing early and late apoptotic cells and DNA fragmentation as detected by gel assays suggest activation of apoptosis following exposure to quercetin (Fig. 8C). Apoptosis involve downregulation of antiapoptotic proteins and upregulation of proapoptotic proteins^50^. Treatment with quercetin resulted in the upregulation of p53, which was consistent with an earlier report^51^. Downregulation of antiapoptotic protein BCL2 and BCL-xL, upregulation of BAX, p-p53, release of CYTOCHROME C, SMAC/DIABLO as well as cleavage of CASPASE 3, CASPASE 9, PARP1 and MCL1 suggested the involvement of intrinsic mitochondrial pathway (Fig. 8C).

Quercetin is suggested to affect multiple cell signaling processes in cancers and various other diseases (Fig. 8C). Heterogeneous nuclear ribonucleoprotein A1 (hnRNPA1) was identified as a target of quercetin in prostate cancer cell line, PC3^52^. Besides, quercetin shown to inhibit hedgehog signaling pathway in prostate cancer^53^. Previous studies also showed that quercetin can enhance TRAIL mediated apoptosis in colon adenocarcinoma cells^54^. Another study suggested set of kinases including ABL1, Aurora-A, B, C as its target^55^. Therefore, it is possible that quercetin treatment might affect multiple targets within the cells, including DNA by direct intercalation as shown in the present study.

In conclusion, quercetin can induce cytotoxicity through multiple routes of action. Our results show that quercetin, a DNA intercalator induces cytotoxicity in cancer cell lines and tumor tissues in mice by activating intrinsic pathway of apoptosis. Therefore, our results suggest that quercetin is a promising candidate in cancer therapeutics.

## Materials and Methods

### Cell lines and cell culture

Human chronic mylegenous leukemia cell line (K562), human acute lymphoblastic leukemia cell line (CEM); human embryonic kidney epithelial cell line (HEK 293T), human breast cancer cell lines (T47D), mouse breast cancer cell lines (EAC) and mouse embryonic fibroblast cell line (MEF-1) were purchased from National Centre for Cell Science, Pune, India. Human B cell leukemia cell line (Nalm6) was a gift from M. Lieber (USA). K562, CEM, T47D and Nalm6 were cultured in RPMI1640 (Sera Lab, USA) with 10% FBS (GIBCO, BRL), 100 U of Penicillin G/ml and 100 μg of streptomycin/ml at 37 °C in humidified atmosphere containing 5% CO_2_. 293T, EAC and MEF were cultured in DMEM containing 10% FBS (GIBCO, BRL), 100 U of Penicillin G/ml and 100 μg of streptomycin/ml.

### Trypan blue exclusion assay

Effect of quercetin and ellagic acid on cell viability was determined on CEM, K562 and Nalm6 cell lines by trypan blue exclusion assay as described^29^,^34^. Cells were seeded at a density of 0.75 × 10^5^ cells/ml for 24 h and treated with different concentrations of quercetin and ellagic acid (0, 10, 50, 100 and 250 μM) for 48 h. IC_50_ value was determined by counting the cells after trypan blue staining using DMSO treated cells as vehicle control. The experiment was repeated a minimum of three independent times and data was presented as bar diagram with standard error mean (SEM).

### MTT assay

Effect of quercetin and ellagic acid on cell proliferation was assessed by MTT assay as described earlier^31^,^56^. Leukemia cell lines (CEM, K562, Nalm6), breast cancer cell lines (EAC and T47D) or normal cell lines (293 T and MEF-1) were seeded (0.75 × 10^5^ cells/ml) for 24 h at 37 °C and then treated with 0, 10, 50, 100 and 250 μM of quercetin or ellagic acid for 48 h and subjected to MTT assay. Experiment was repeated a minimum of three times independently, each with duplicate sets of reaction and presented as bar diagram along with standard error mean (SEM).

### Cell cycle analysis by flow cytometry

Cell cycle analyses was performed in Nalm6 cell line as described^29^,^33^. A time course experiment was conducted on Nalm6 cells following treatment with quercetin (20 μM) for 8, 16 and 24 h. Besides Nalm6 cells were also treated with increasing concentrations of quercetin (0, 10, 50, 100, 250 μM) for 48 h. In both the experiments cells were seeded at 0.75 × 10^5^ cells/ml, cells were harvested after quercetin treatment, washed, fixed and incubated with RNase A. Cells were then stained with propidium iodide (50 μg/ml) and readings were acquired in flow cytometer (BD Biosciences FACS Calibur, USA). A minimum of 10,000 cells were acquired per sample and data was analyzed by using WinMDI 2.9 software. Experiments were repeated a minimum of two independent times and data is presented along with error bars.

### Circular Dichroism (CD)

DNA binding ability of quercetin was studied using CD as described earlier^31^. Quercetin (0, 50, 100, 150 μM) was incubated with sheared calf thymus (CT) DNA in buffer (10 mM Tris HCl, 15 mM NaCl) for 1 h and spectra were recorded at a wavelength of 210–320 nm on JASCO J-810 spectropolarimeter. Spectra for buffer alone and buffer with corresponding concentrations of quercetin without DNA were subtracted from the experimental data. CD spectra of ethidium bromide (0, 10, 20 μM) incubated with CT DNA served as a positive control.

### DNA mobility shift assay

The assay was performed as described earlier^31^. 100 ng of supercoiled and linearized pUC18 plasmid was incubated with quercetin (0, 10, 50, 100, 150, 200, 250 μM) at room temperature for 1 h and resolved on agarose gel. For further details refer supplementary methods.

### Animal and ethics statement

Mice were maintained in strict accordance with the principles and guidelines of the ethical committee for animal care of Indian Institute of Science (IISc) in accordance with Indian National Law on animal care and use. The experiments designed for the present study were approved by Institutional Animal Ethical Committee (Ref. CAF/Ethics/289/2012) IISc, Bangalore, India. For other details refer supplementary methods.

### Preparation of EAC cells and tumor induction

Fixed number of ehrlich ascites carcinoma cells (1 × 10^6^ cells/animal) were injected in the peritoneal cavity of donor mouse and allowed to multiply. The cells were withdrawn, diluted in 1X PBS, counted and re-injected to the right thigh of the experimental animals (1 × 10^6^ cells/animal) for developing solid tumor.

### Evaluation of anticancer effects of quercetin and ellagic acid on tumor development

Total of 15 mice were taken in a batch, of which 10 were injected with EAC cells in their right thigh to develop solid tumor. 5 mice served as no tumor control, which did not receive quercetin or ellagic acid treatment (Group I). 10 mice with tumor were divided into two groups based on size of the tumor so that mean tumor size of each group remain equal, one of which served as tumor control and received no treatment (Group II), while quercetin (1 mg/kg) or ellagic acid (3 mg/kg) was administered to the other by oral gavage method on 12^th^ day of EAC injection when small tumor was visible (Group III). This group received 6 doses of compound every 3 days. The study was repeated for three independent batches. The dose selected for final study was based on a pilot study conducted using different doses of quercetin and ellagic acid (1, 3 and 10 mg/kg).

Tumor volume was determined by measuring tumor diameter after every 4 days for each animal using vernier calipers as described^24^,^28^,^31^. Tumor volume was calculated by using the formula, V = 0.5ab^2^ where ‘a’ and ‘b’ represents major and minor diameter, respectively^31^. After 30^th^ and 45^th^ days one animal was sacrificed from each group by cervical dislocation and tissues were fixed separately for normal, control and treated animals from multiple batches.

### Determination of effect of quercetin on survival of tumor animals

The % survival of quercetin treated mice was calculated and compared with control animals. Five animals bearing tumor were taken in each control and quercetin treated groups. The study was done in two independent batches. The death pattern of control and treated animals was reported and percentage increase in life span was calculated using the formula [(T-C)/C] × 100, where T indicates the time of survival of treated animals while C represents survival time of control animals^24^,^28^,^46^. The treated animals were monitored for at least 250 days.

### Histological evaluation

Tumor tissues from control and quercetin treated animals were collected. Tissues were embedded in paraffin wax and sections of 5 μm were cut in rotary microtome (Leica Biosystems, Germany). Sections were de-paraffinized and hematoxylin and eosin staining was done as described^23^,^57^. Sections were evaluated for structural changes using light microscope (Carl Zeiss, Germany) and images were captured.

### TUNEL assay

Terminal deoxynucleotidyl transferase (TdT) dUTP Nick-End Labeling (TUNEL) assay was performed using DNA fragmentation Detection Kit (Calbiochem, USA). For further details refer supplementary methods.

### Immunohistochemistry (IHC)

Immunohistochemical staining was performed on quercetin treated tissues sections as described before^31^,^57^. For further details refer supplementary methods.

### JC1 staining to detect change in mitochondrial membrane potential

5,5’,6,6’-tetrachloro-1,1’,3,3’ tetraethylbenzimidazolylcarbocyanine iodide/chloride (JC-1), a fluorescent carbocyanin dye, was used to check the transmembrane potential (ΔψM) of mitochondria^25^. For further details refer supplementary methods.

### Annexin V-FITC/propidium iodide (PI) staining for apoptotic stages

Annexin V-FITC/PI staining was carried out to detect early and late apoptotic cellular stages, as described^34^. For further details refer supplementary methods.

### Immunoblotting

Nalm6 cell lines were seeded at a density of 0.75 × 10^5^ cells/ml for 24 h. Following treatment with quercetin (0, 10, 20 μM, for 24 h), cells were harvested, and lysate was prepared in RIPA buffer (25 mM Tris (pH 7.6), 150 mM NaCl, 1% NP-40, 1% sodium deoxycholate and 0.1% SDS) as described^34^,^57^. Protein concentration was determined by Bradford assay. Approximately, 30 μg lysate was resolved on 10–12% SDS-PAGE and transferred onto the PVDF membrane (Millipore, USA). Membrane was blocked and incubated with respective primary antibodies (BCL2, BAX, MCL1, CASPASE 9, activated CASPASE 3, CYTOCHROME C, p53, p-p53, SMAC/DIABLO, BCL-xL, PARP1, Tubulin) at 4 °C overnight. Blots were washed, incubated with HRP-conjugated secondary antibodies, developed using chemiluminescent solution (Immobilon^TM^ western, Millipore, USA) and scanned by using gel documentation system (ImageQuant LAS4000, GE, USA). The experiment was repeated a minimum of three independent times.

### DNA fragmentation assay

Nalm6 cells were treated with quercetin (0, 10, 50, 100, 250 μM) as described above and DNA fragmentation analysis was performed^58^. For further details refer supplementary methods.

### Statistical analysis

Data was expressed as mean ± SEM for normal and experimental animals. Statistical analysis was performed by two way ANOVA and student’s t-test using software Graph pad prism 5.1. Values were considered significant when P ≤ 0.05. Kaplien-Meier survival curve was also plotted using Graph pad prism 5.1.

## Additional Information

**How to cite this article**: Srivastava, S. *et al*. Quercetin, a Natural Flavonoid Interacts with DNA, Arrests Cell Cycle and Causes Tumor Regression by Activating Mitochondrial Pathway of Apoptosis. *Sci. Rep*. **6**, 24049; doi: 10.1038/srep24049 (2016).

## Supplementary Material

Supplementary Information
