# Supplementary material for: Quercetin, a Natural Flavonoid Interacts with DNA, Arrests Cell Cycle and Causes Tumor Regression by Activating Mitochondrial Pathway of Apoptosis
Source: Sci Rep. 2016 Apr 12;6:24049. doi: 10.1038/srep24049 (PMC4828642; doi:10.1038/srep24049)
Supplement: Supplementary Information [file srep24049-s1.pdf]

Quercetin, a Natural Flavonoid Interacts with DNA, Arrests Cell Cycle and Causes Tumor Regression by Activating Mitochondrial Pathway of Apoptosis

Shikha Srivastava[1], Ranganatha R. Somasagara [1], Mahesh Hegde[1], Mayilaadumveettil Nishana[1], Satish Kumar Tadi[1], Mrinal Srivastava[1], Bibha Choudhary[2] and Sathees C. Raghavan[1*]

Supplementary Information

Supplementary Methods

Chemicals and Reagents

All the chemicals used in the present study were purchased from Sigma Aldrich (St. Louis, MO, USA) or Sisco Research Laboratories (India). Antibodies were from Santa Cruz Biotechnology (USA), BD Biosciences (USA) and Cell Signaling Technology (USA). Purified quercetin and ellagic acid (Suppl. Fig. 1) were purchased from Sigma Aldrich (USA). Stock solutions of quercetin and ellagic acid were prepared in DMSO and stored at 4°C till use. When required, dilutions were done in autoclaved miiliQ water.

DNA mobility shift assay

The assay was performed as described earlier [1]. 100 ng of supercoiled and linearized pUC18 plasmid was incubated with increasing concentrations of quercetin (0, 10, 50, 100, 150, 200, 250 μM) at room temperature for 1 h. Ethidium bromide (0, 2.5, 10, 25 μM, at 37°C for 15 min) served as positive control in both the cases. Samples were resolved on 1.2% agarose gel (30 V), visualized under UV transilluminator and images were captured using gel documentation system (Uvitec, Cambridge, UK).

Animal and ethics statement

Female Swiss albino mice weighing 18-22 g and 6-8 weeks old were purchased from central animal facility of IISc, India for the present study. The mice were maintained under controlled conditions of temperature and humidity with 12 h light and dark cycle. They were housed in polypropylene cages and standard pellet diet (Agro Corporation Pvt. Ltd. Bangalore, India) was provided along with water ad libitum. The standard pellet diet was composed of 21% protein, 4% crude fiber, 5% lipids, 3.4% glucose, 2% vitamins, 1% calcium, 0.6% phosphorus, 8% ash and 55% nitrogen-free extract (carbohydrates).

TUNEL assay

Terminal deoxynucleotidyl transferase (TdT) dUTP Nick-End Labeling (TUNEL) assay was performed on histological sections (5 μm) of quercetin treated tumor tissue and untreated

tumor control after 30th day of treatment for the detection of DNA strand breaks occurred during apoptosis. Sections were de-paraffinized and assay was performed by using the TUNEL assay kit (DNA fragmentation Detection Kit, Calbiochem, USA). Briefly, sections were washed in PBS (1X) and treated with proteinase K for permeabilization of the specimen. Labeling was done with dUTP at nicked DNA ends with the help of TdT enzyme at 37°C for 1 h, 30 min followed by DAB staining. Methyl green was used as a counterstain. Sections were observed under light microscope (Carl Zeiss, Germany) and images were captured. Percentage of TUNEL positive nuclei were determined by counting a minimum of five different randomly selected sections and shown as bar diagram with SEM.

Immunohistochemistry (IHC)

Sections with thickness 5 µm from control tumor and quercetin treated tumor tissues embedded in paraffin wax were cut in rotary microtome and deparaffinized as described earlier [1, 2]. Sections were then rehydrated and treated with 3% H_2O_2. Antigen retrieval was performed in 0.01% sodium citrate buffer followed by blocking (0.1% BSA and 10% FBS in PBST) for 1 h at room temperature. Following incubation in primary antibody (Ki-67, p53 and p-p53; overnight at 4°C), slides were washed with PBST and incubated with biotinylated secondary antibodies (1:200) for 2 h at room temperature. Slides were washed in PBST, and incubated in streptavidin-HRP (1:500) at room temperature. Following washing, slides were developed using DAB+ H_2O_2, counterstained with hematoxylin and mounted using DPX. Images were captured using light microscope (Carl Zeiss, Germany). Tumor sections were quantified by counting DAB positive cells (brown colored) from a minimum of five different randomly selected fields and the percentage of positive cells were determined by using the formula, number of positive cells X 100 / Total number of cells.

Calcein-A-propidium iodide assay

Nalm6 cells were treated with quercetin (0, 20 and 50 µM) for 48 h. Cells were harvested, washed with ice-cold PBS and incubated with calcein-A (0.16 µM) and propidium iodide (0.66 µg/µl) for 20 min at room temperature in dark and samples were analyzed by flow cytometry.

JC1 staining to detect change in mitochondrial membrane potential

5,5',6,6'-tetrachloro-1,1',3,3' tetraethylbenzimidazolylcarbocyanine iodide/chloride (JC-1), a fluorescent carbocyanin dye, was used to check the transmembrane potential (ΔψM) of mitochondria [6]. Briefly, Nalm6 cells were seeded (0.75x10^5 cells/ml) in 6-well plate and incubated at 37°C in the presence of 5% CO_2 for 24 h. Following quercetin treatment (10, 20, 50, 100 μM, 12 h), cells were harvested, washed with ice cold PBS and incubated with JC1 dye (0.5 μM) for 20 min at 37°C in dark. Cells were washed and samples were analyzed by flow cytometer after applying appropriate gates. A shift from red (590 nm, FL-2 channel) to green (529 nm, FL-1 channel) fluorescence was observed with the change in mitochondrial membrane potential. 2,4 dinitro phenol (2,4-DNP) was used as positive control.

Annexin V-FITC/propidium iodide (PI) staining for apoptotic stages

Annexin V-FITC/PI staining was carried out to detect early and late apoptotic cellular stages, as described [7]. Nalm6 cells were treated with quercetin (0, 20 and 50 μM, for 6, 12, 18, 24, 48 h), cells were harvested, washed with ice-cold PBS and resuspended in binding buffer (Calbiochem, USA). Annexin V-FITC (0.2 μg/μl) and PI (0.05 μg/μl) were added and incubated on ice for 45 min and samples were analyzed by flow cytometry.

Confocal microscopy

Annexin V-FITC/PI stained Nalm6 cells were prepared as described above after treatment with quercetin (0, 20 and 50 μM, for 48 h). Cells were mounted on glass slides and images were captured under inverted Zeiss confocal laser-scanning microscope (Ziess Meta 510 LSM; Carl Zeiss, Jena, Germany).

Detection of intracellular ROS by flow cytometry

Reactive oxygen species (ROS) is produced by each cell at intrinsic level, which may get enhanced after treatment with some cytotoxic compounds. 2, 7-dichloro-dihydro fluorescein diacetate (H_2DCFDA, Sigma, USA) is a cell permeable fluorescent dye, which gets oxidized by ROS and converted to fluorescent dichlorofluorescein [8]. Following quercetin treatment (25 μM), Nalm6 cells were harvested at different time points (10 min, 30 min, 1, 2, 8 and 12 h). Cells

were stained with H_2DCFDA (5 μM) and incubated at 37°C for 20 min in dark and analyzed with flow cytometry. Cells treated with H_2O_2 served as positive control.

DNA fragmentation assay

Nalm6 cells were treated with quercetin (0, 10, 50, 100, 250 μM) as described above and DNA fragmentation analysis was performed [9]. Cells were harvested, washed with ice-cold PBS and incubated in DNA lysis buffer (10 mM Tris, [pH 8.0], 100 mM NaCl, 0.25% Triton X-100, 1 mM EDTA) along with proteinase K (60 μg/ml) and RNase A (100 μg/ml) at 55°C for 4 h. Extraction was done using phenol-chloroform (1:1) and chloroform. DNA was precipitated with 1/10 volume of 3 M sodium acetate and equal volume of isopropanol. Quantitation was done using Nano drop spectrophotometer (Nanodrop, ND-2000). 7 μg DNA was loaded in each lane along with DNA ladder, resolved on 2% agarose gel at 50 V for 5 h and visualized in UV transilluminator. Image was taken by using gel documentation system for analysis. 100 μM 5-fluorouracil (5-FU) acted as a positive control for the assay.

Supplementary References

1. Srivastava, M. et al. An inhibitor of nonhomologous end-joining abrogates double-strand break repair and impedes cancer progression. *Cell* **151**, 1474-1487 (2012).
2. Sharma, S., Choudhary, B. & Raghavan, S.C. Efficiency of nonhomologous DNA end joining varies among somatic tissues, despite similarity in mechanism. *Cell Mol Life Sci* **68**, 661-676 (2011).
3. Nishana, M. & Raghavan, S.C. A non-B DNA can replace heptamer of V(D)J recombination when present along with a nonamer: implications in chromosomal translocations and cancer. *Biochem J* **448**, 115-125 (2012).
4. Naik, A.K., Lieber, M.R. & Raghavan, S.C. Cytosines, but not purines, determine recombination activating gene (RAG)-induced breaks on heteroduplex DNA structures: implications for genomic instability. *J Biol Chem* **285**, 7587-7597 (2010).
5. Raghavan, S.C., Swanson, P.C., Ma, Y. & Lieber, M.R. Double-strand break formation by the RAG complex at the bcl-2 major breakpoint region and at other non-B DNA structures in vitro. *Mol Cell Biol* **25**, 5904-5919 (2005).
6. Chiruvella, K.K. et al. Methyl angolensate, a natural tetranortriterpenoid induces intrinsic apoptotic pathway in leukemic cells. *FEBS Lett* **582**, 4066-4076 (2008).
7. Kavitha, C.V. et al. Novel derivatives of spirohydantoin induce growth inhibition followed by apoptosis in leukemia cells. *Biochem Pharmacol* **77**, 348-363 (2009).
8. Chiruvella, K.K. & Raghavan, S.C. A natural compound, methyl angolensate, induces mitochondrial pathway of apoptosis in Daudi cells. *Invest New Drugs* **29**, 583-592 (2011).
9. Sahu, U. et al. A Novel Anticancer Agent, 8-Methoxypyrimido[4',5':4,5]thieno(2,3-) Quinoline-4(3H)-One Induces Neuro 2a Neuroblastoma Cell Death through p53-Dependent, Caspase-Dependent and -Independent Apoptotic Pathways. *PLoS One* **8**, e66430 (2013).

Supplementary Figure Legends

Supplementary Figure 1. Chemical structures of quercetin and ellagic acid.

Supplementary Figure 2. Determination of cytotoxic effect of quercetin in breast cancer and normal cells. Cytotoxic effect of quercetin was analysed on (**A**) human breast cancer cell line, T47D, mouse breast cancer cell line, EAC, human embryonic kidney epithelial cell line, HEK293T and (**B**) mouse embryonic fibroblast, MEF-1. Cell lines were treated with different concentrations of quercetin as indicated for 48 h to determine cytotoxicity. Effect of ellagic acid was also tested and shown in the case of EAC cells. In all cases, cytotoxicity was determined by MTT assay. 'ns' denotes 'not significant' while '*' represents significance (*P< 0.05, ** P< 0.01, ***P< 0.001).

Supplementary Figure 3. Detection of dead and live cells after treatment of quercetin by calcein-A/PI staining. Nalm6 cells were treated with quercetin (0, 20 and 50 μM) and subjected to calcein-A/PI staining. Cells in lower left quadrant were negative for both calcein and PI, lower right quadrant shows cells stained with calcein alone (live cell population), left upper quadrant shows dead cells (stained with PI alone) while upper right quadrant shows cells positive for both calcein and PI.

Supplementary Figure 4. Detection of intracellular ROS production following quercetin treatment in Nalm6 cells. A-B. Nalm6 cells were treated with 25 μM quercetin, incubated for **A.** early time points (10, 30 min, 1 and 2 h), and **B**. late time points (8 h and 12 h), harvested and analysed for ROS production by flow cytometry. H_2O_2 treated cells were served as positive control. VC stands for DMSO treated vehicle control.

Supplementary Figure 5. Confocal images of annexin V-FITC/PI stained apoptotic cells. Nalm6 cells treated with quercetin (0, 20 and 50 μM for 48 h) were subjected to annexin V-FITC/PI staining. Most of the control cells were negative for both annexin V/PI staining showing cellular integrity. Cells in early apoptosis stained with annexin V alone (green in color; upper left), while late stage apoptotic cells were stained with both annexin V-PI (green and red, lower

right). In each image upper right quadrant indicate bright field (DIC image) and lower left represents cells stained with PI alone (red).

Supplementary Figure 6. Quantitation showing effect of quercetin on expression of apoptotic proteins. Quantitation of bands observed following western blotting is shown as bar diagram and standard error mean (SEM) is indicated based on three independent repeats. 'ns' indicates 'not significant' while '*' represents significance (*P< 0.05, ** P< 0.01). For other details refer Figure 8 legend.

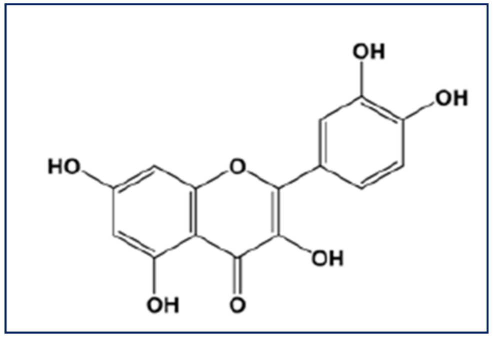

QUERCETIN

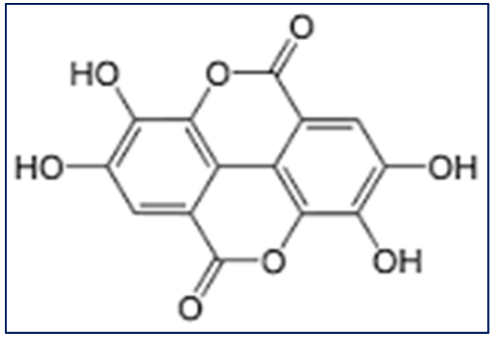

ELLAGIC ACID

Suppl. Figure 1

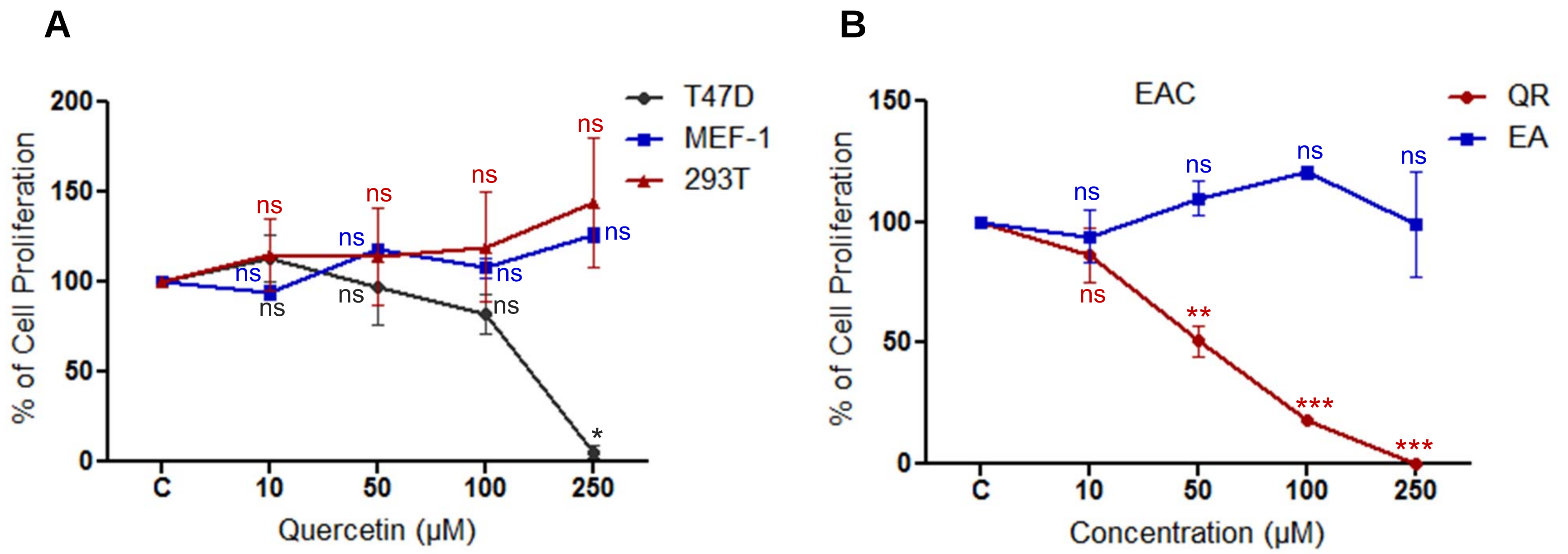

Suppl. Figure 2

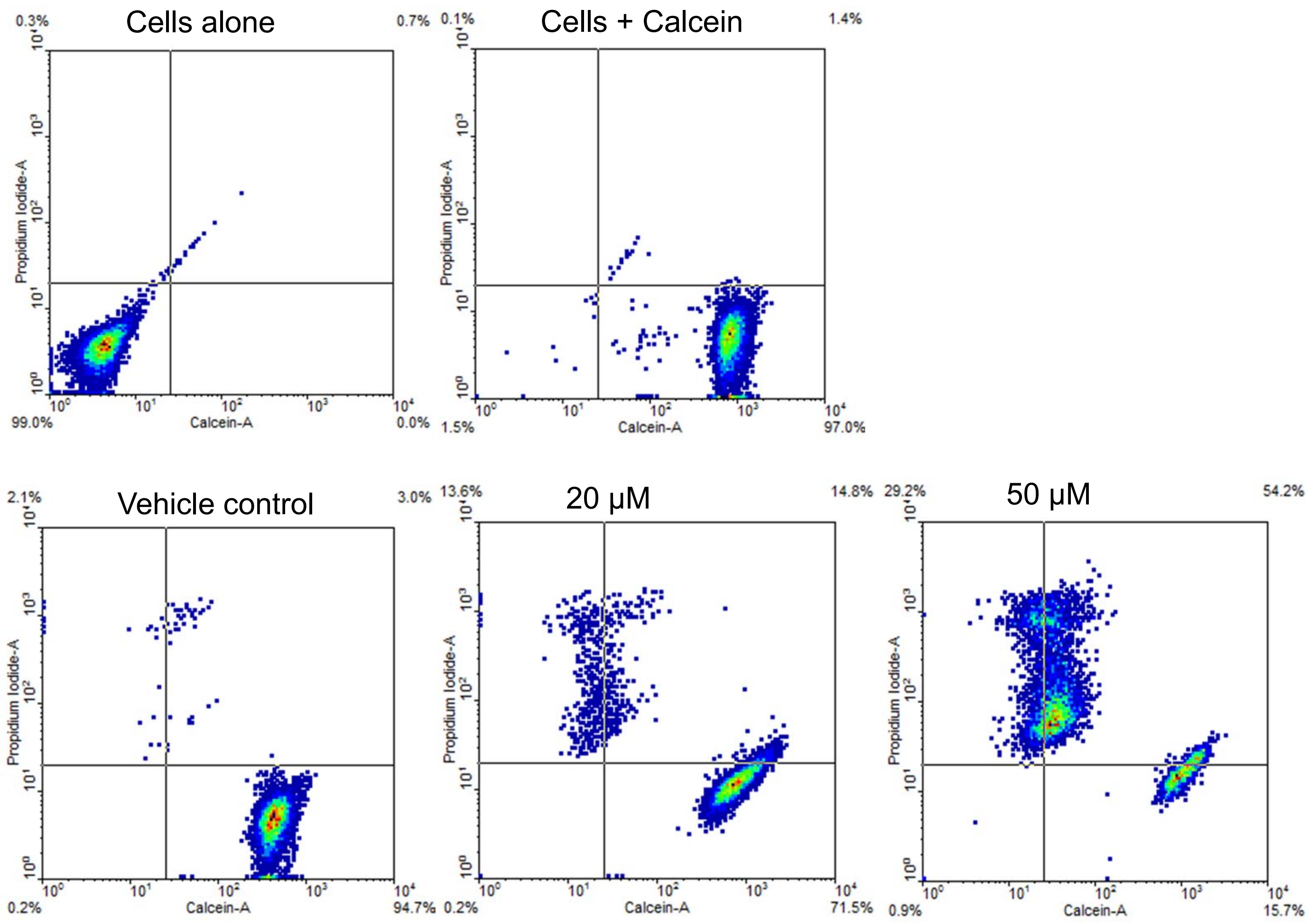

Suppl. Figure 3

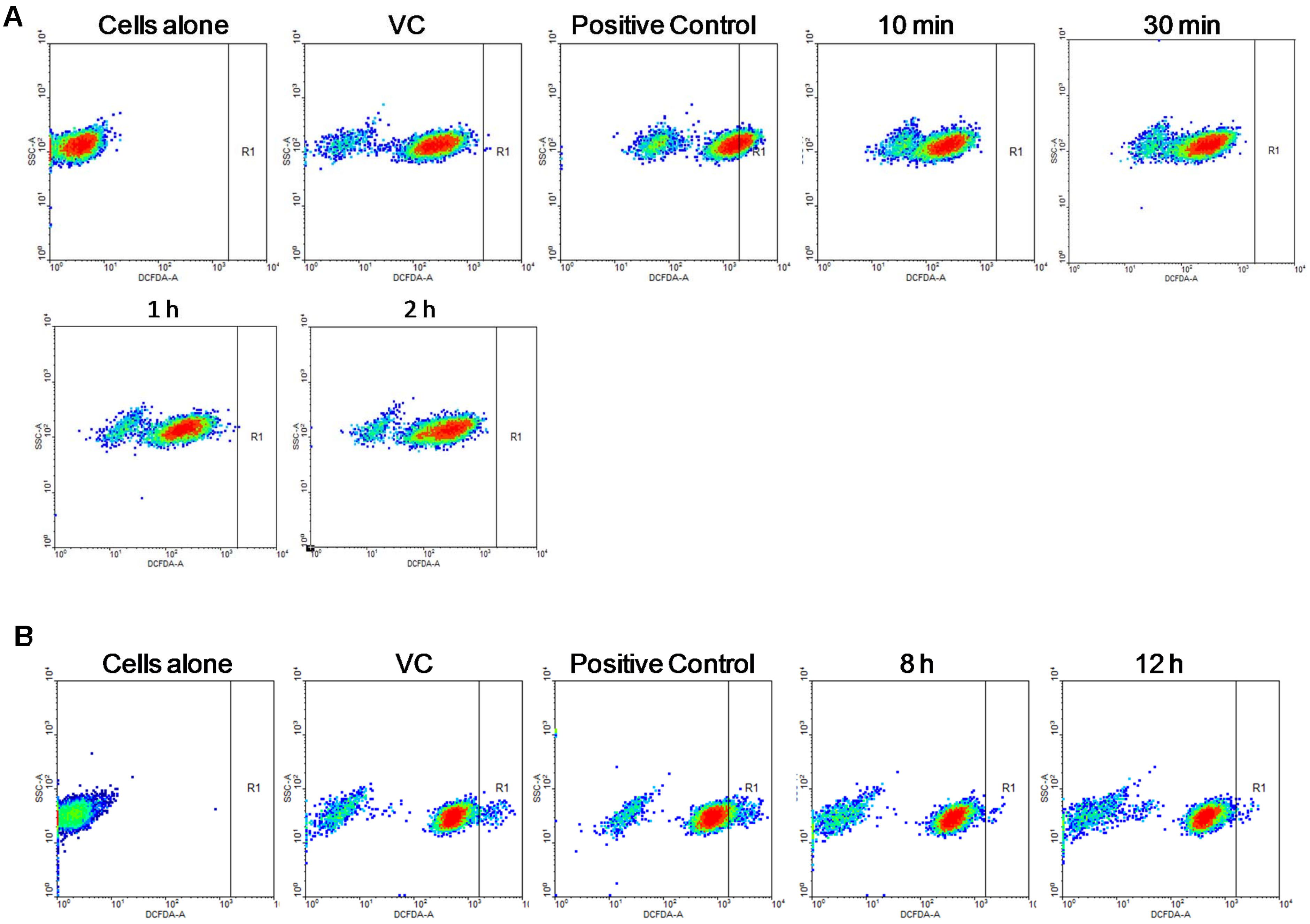

Suppl. Figure 4

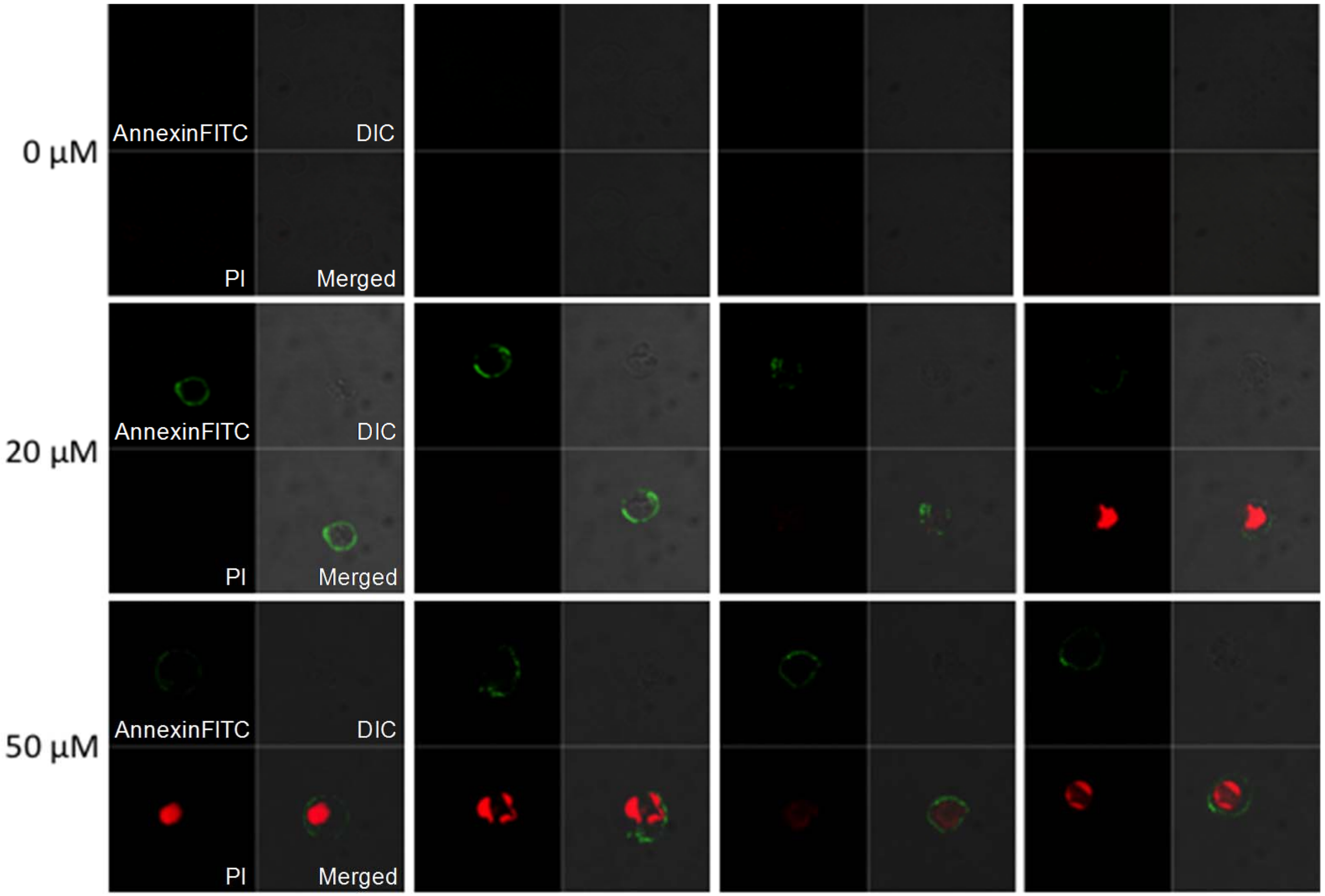

Suppl. Figure 5

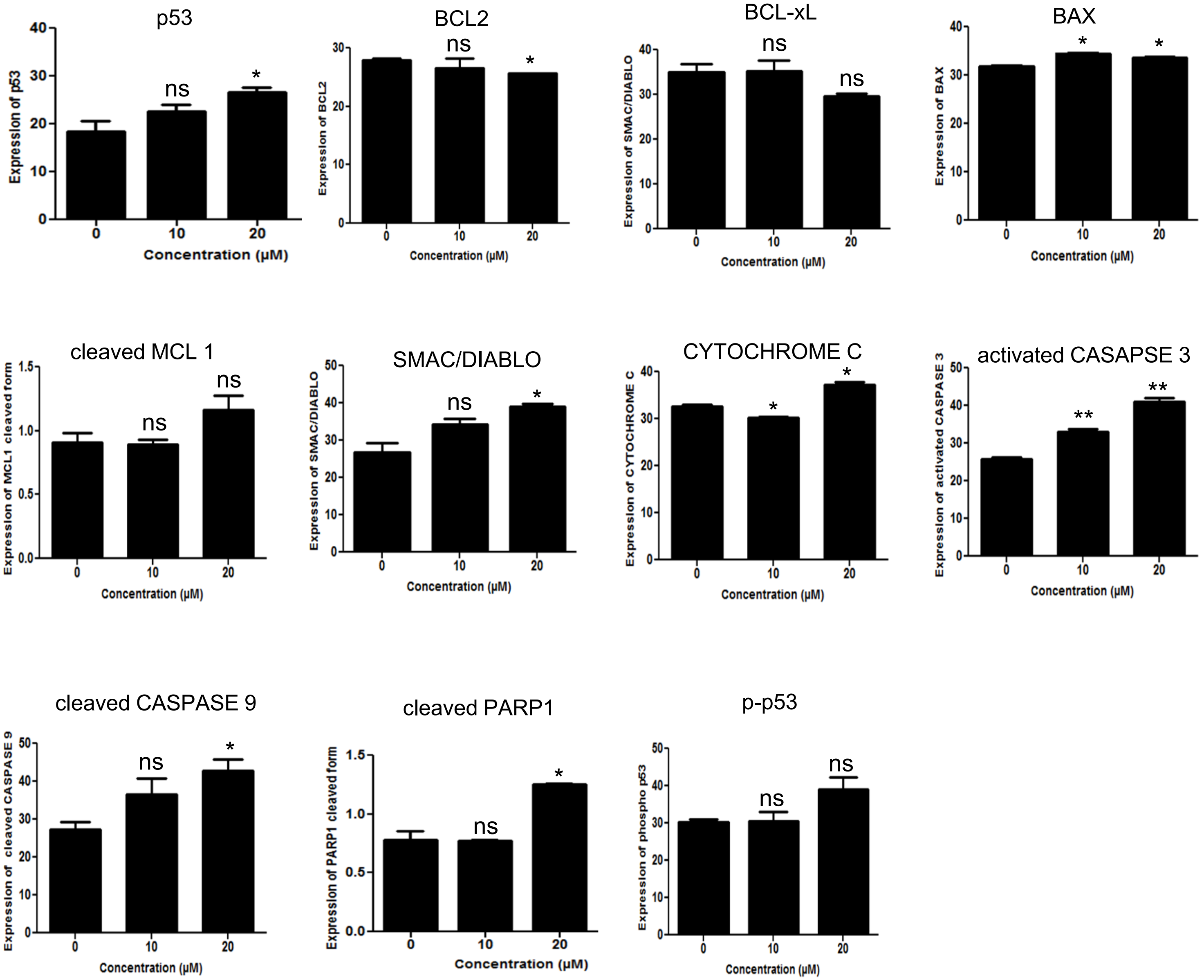

Suppl. Figure 6
